# Adaptation and Validation of the Foot Function Index-Revised Short Form into Polish

**DOI:** 10.1155/2017/6051698

**Published:** 2017-11-27

**Authors:** Radosław Rutkowski, Małgorzata Gałczyńska-Rusin, Małgorzata Gizińska, Marcin Straburzyński-Lupa, Agata Zdanowska, Mateusz Wojciech Romanowski, Wojciech Romanowski, Elly Budiman-Mak, Anna Straburzyńska-Lupa

**Affiliations:** ^1^Department of Physical Therapy and Sport Recovery, Poznan University of Physical Education, Królowej Jadwigi 27/39, 61-871 Poznan, Poland; ^2^Department of Gerostomatology and Oral Pathology, Poznan University of Medical Sciences, Bukowska 70, 60-812 Poznan, Poland; ^3^Department of Urology, Hospital in Puszczykowo, Kraszewskiego 11, 62-040 Puszczykowo, Poland; ^4^Rheumatological Centre in Śrem, Mickiewicza 95, 63-100 Śrem, Poland; ^5^Department of Rheumatology and Rehabilitation, Poznan University of Medical Sciences, Poznan, Poland; ^6^Center of Innovation for Complex Chronic Healthcare (CINCCH), Hines VA Hospital, 5000 South 5th Ave, Hines, IL 60141-3030, USA; ^7^Department of Medicine, Stritch School of Medicine, Loyola University of Chicago, Maywood, IL 60513, USA

## Abstract

**Purpose:**

The aim of the present study was to adapt the Foot Function Index-Revised Short Form (FFI-RS) questionnaire into Polish and verify its reliability and validity in a group of patients with rheumatoid arthritis (RA).

**Methods:**

The study included 211 patients suffering from RA. The FFI-RS questionnaire underwent standard linguistic adaptation and its psychometric parameters were investigated. The enrolled participants had been recruited for seven months as a convenient sample from the rheumatological hospital in Śrem (Poland). They represented different sociodemographic characteristics and were characterized as rural and city environments residents.

**Results:**

The mean age of the patients was 58.9 ± 10.2 years. The majority of patients (85%) were female. The average final FFI-RS score was 62.9 ± 15.3. The internal consistency was achieved at a high level of 0.95 in Cronbach's alpha test, with an interclass correlation coefficient ranging between 0.78 and 0.84. A strong correlation was observed between the FFI-RS and Health Assessment Questionnaire-Disability Index (HAQ-DI) questionnaires.

**Conclusion:**

The Polish version of FFI-RS-PL indicator is an important tool for evaluating the functional condition of patients' feet and can be applied in the diagnosis and treatment of Polish-speaking patients suffering from RA.

## 1. Introduction

Foot disorders are currently very common; they significantly limit patients' activity and worsen their quality of life (QoL). It is assumed that the incidence of these disorders varies between 10% and 24% and in most cases concerns older people [1, 2]. Patients suffering from rheumatoid arthritis (RA) deserve special attention. Based on previous studies, in most cases, arthritis-related damage (arthropathy) of the foot joints occurs within 10 years of disease onset [3–5]. About 15% of patients report that the first symptoms are located within the feet [4]. These changes can have a negative impact on patients' physical and psychological functioning and therefore worsen their quality of life (QoL). Arthropathy can be located only in the forefoot or can affect the whole foot. Pain associated with arthropathy may persist even during the remission period of the disease, thus affecting the personal choice of footwear and clothing [5].

Questionnaires evaluating the QOL, mobility (or daily living activity), and disease progression are clinically significant [6]. There are many tools that have been successfully validated and are applied in many countries [1, 4, 7, 8].

One such questionnaire is the Foot Function Index (FFI). It was established in 1991 based on clinical observations and experiments by three American authors: Budiman-Mak, Conrad, and Roach [9]. At the beginning, the questionnaire consisted of 23 questions grouped into 3 subcategories, in which subjective impressions concerning the impact of foot disorder on pain, difficulty, and activity limitation were quantified [9]. The questionnaire was revised in 2006, due to comments raised by clinicians and researchers, and the FFI-R (R: revised) was established. Questions evaluating the psychosocial aspects of activity and quality of life, depending on the condition of the feet, have been added [1]. Currently, there are 2 variants of the questionnaire: FFI-RL (long form), a long version consisting of 4 parts including 68 questions in total, and FFI-RS (short form), a short version containing 34 questions. Both versions take 5 subcategories into consideration: pain, stiffness, difficulty, activity limitation, and social aspects. According to the authors, both the long and short versions have sound psychometric measures [1].

The aim of this study was to translate the original short form of the FFI-RS questionnaire into Polish and to verify its reliability and validity in a group of Polish-speaking patients suffering from RA.

## 2. Materials and Methods

The study involved 211 patients. Participant inclusion criteria were adults with RA diagnosed according to ACR criteria from 2010 [10] and arthritis-related pain and/or swelling of the joints within the feet in addition to agreeing to participate voluntarily in the study and giving prior informed consent. All participants were recruited as a convenient sample from the rheumatological hospital in Śrem, Poland, and represented different sociodemographic characteristics. They were characterized as rural and city environments near hospital place. The study procedure lasted seven months. Cognitive, proprioceptive, sensory impairment, foot fractures, and surgery during the last six months were exclusion criteria for this study.

Each participant was coached to correctly fill the questionnaire.

*Ethical Considerations*. This study was approved by the Research Ethics Committee at Poznan University of Medical Sciences, under protocol number 183/14, and all the participants were informed and signed the free consent statement. The permission to translate was also granted by Dr. Elly Budiman-Mak, the primary author of the original FFI-R short form.

## 3. Scoring Method

The FFI-RS questionnaire consists of 34 questions grouped into 5 subcategories: pain (7 questions), stiffness (7 questions), difficulty (11 questions), activity limitation (3 questions), and social issues (6 questions). The answers are represented by a 4-level Likert scale. The numerical 5 is not a Likert scale; it is an option that the particular question is not applicable. The scoring of the FFI-R is based on the assumption that the severity of impairment in foot function is determined by the combination of pain, stiffness, difficulty, activity limitation, and psychosocial scales. A score is derived for each item by marking the patient experience on the choices displayed on a Likert scale. For example, on the pain subscale, grade 1 corresponds to no pain and 4 corresponds to worst pain imaginable. The numerical 5 is not used as a score but is used to indicate that the subscale question is not applicable for the study subject (participant). To obtain a subscale score, the item scores for a subscale are added and divided by the maximum total possible for the subscale items that the patient indicated were applicable, after which they are multiplied by 100.

This method of scoring was applied to all subscales.

The FFI-R short form score will be the average of the five subscale scores.

The total scores of each subscale, pain, stiffness, difficulty, activity limitation, and psychosocial, will be summed and then divided by 5 [11].

## 4. The Translation Process of the Polish FFI-RS (FFI-RS-PL)

**Figure 1:**
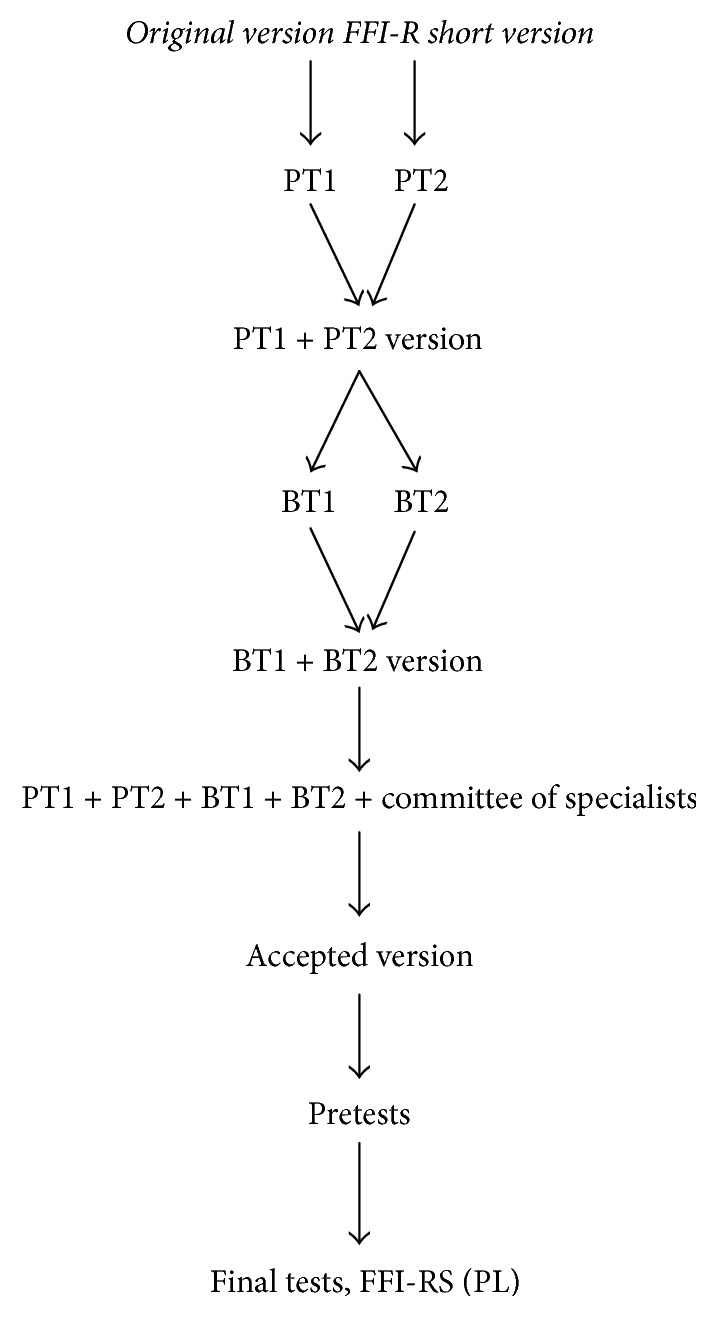
Stages of FFI-R short version questionnaire translation process into Polish.

The translation process started after obtaining the permission from the authors of the original FFI-R short form to translate FFI-RS into Polish. The process is depicted in Figure 1. The translation was conducted according to generally accepted guidelines [12]. The stages included translating the original version of the questionnaire into Polish (PT-Polish translation) by two independent translators (a health professional and an English lecturer), who were fluent in English and whose native language was Polish (PT1, PT2). Afterwards an agreed version was established (PT1 + PT2 version). The next step was to establish a back-translation (BT) from Polish into English. The back-translation was conducted by two persons (BT1 and BT2), who were native English speakers and were blinded to the original English version of the questionnaire. After verifying both of the translations, the final version was established (BT1 + BT2 version). A team of specialists consisting of rheumatologists and physiotherapists analyzed the appropriateness of the medical wording (the committee of specialists analysis). The entire translation process was supervised by a main researcher who did not participate in the translation process.

The accepted version (see Figure 1) was tested on a group of participants (*N* = 211), whose native language was Polish, with the minimum basic educational level of primary school level. During the test, each participant could write a comment about the experience in using FFI-RS-PL. Participants had no difficulty in understanding or answering the questionnaires. The average time needed to complete the test was 10 minutes. The test was rated positively regarding the transparency of all of the questions and their appropriateness, clarity, length, and usefulness. A final Polish version of the Foot Function Index-Revised Short Form-Polish Version (FFI-RS-PL) was accepted (see Supplementary Material).

## 5. The Compatibility Characteristics of FFI-RS-PL

In order to perform a test of correlation and statistical analysis with FFI-RS-PL questionnaire, each participant had to fill out the HAQ-DI (Health Assessment Questionnaire-Disability Index) and assess the pain level using the visual analogue scale (VAS pain scale). Moreover, the duration of the disease and the number of swollen and tender joints were recorded for each participant individually.

### 5.1. Health Assessment Questionnaire-Disability Index

To examine physical function, the Health Assessment Questionnaire-Disability Index (HAQ-DI) was used. This questionnaire evaluates patients' health functional status and correlates it with other biochemical and clinical measures, comorbidities, healthcare resource utilization and cost estimations, and mortality. The HAQ-DI is composed of 20 detailed questions about daily activities, divided into 8 categories: dressing and taking care of appearance, arising, eating, walking, hygiene, reaching, gripping, and daily life activities. All respondents assessed their own difficulty in carrying out each of the activities on a scale from 0 to 3 (0 means no difficulty in performing the task and 3 means the task was impossible to perform) [13].

### 5.2. Disease Activity Score 28

DAS 28 is an important and very often used outcome for clinical practice and research all over the world in patients with rheumatoid arthritis. Published thresholds define absolute DAS 28 scores representing remission (<2.6), mild (≤3.2), moderate (>3.2), or severe (>5.1) disease activity [14, 15].

### 5.3. The Visual Analogue Pain Scale Was Used to Assess Pain Severity

The VAS pain scale results were obtained by measuring the distance in millimeters from the beginning of the scale to the position selected by the patient from 0 to 100 mm where 0 is “no pain” and 100 is “the worst possible pain” [16]. Structurally, we find the VAS pain scale to be a simple tool to use by anyone cognitively capable of understanding the parameters and responding to clinician's instructions. Indeed its popularity is frequently attributed to the ease and convenience of the VAS pain scale in a fast-paced clinical setting [16].

For the evaluation of the functional assessment of both feet, each patient defined the number of swollen and tender metatarsophalangeal (MTP) joints [14]. Each subject underwent a 4-degree assessment of overall functional condition (ACR revised classification of functional status in RA), which was performed by a rheumatologist. On this basis, the patients' functional condition and capability of independent living were determined. Patients were classified according to the following criteria:

 
*Class I*. They were completely able to perform usual activities of daily living (self-care, vocational, and avocational).
 
*Class II*. They were able to perform usual self-care and vocational activities, but limited avocational activities.
 
*Class III*. They were able to perform usual self-care activities, but limited vocational and avocational activities.
 
*Class IV*. They had limited ability to perform usual self-care, vocational, and avocational activities [17].

## 6. Statistical Analysis

The reliability of the FFI-RS questionnaire was assessed by analyzing its internal consistency using Cronbach's alpha coefficient and the test-retest reliability method. Subsequently, the diagnostic, convergent, and divergent validity were assessed. Diagnostic validity, that is, the coherence of the test with external criteria, was evaluated by checking the association between the FFI-RS scores and functional condition, duration of RA, and pain location within the MTP joints. It was assumed that patients with a higher FFI-RS score would be characterized by worse functional condition and longer duration of the disease. It was also assumed that there would be a statistically significant difference in pain scale score between patients with pain located within the MTP and patients with pain in another location. Convergent validity was assessed by checking the correlation between scores of FFI-RS and HAQ-DI scale, VAS pain scale, and HAQ-DI subscale concerning walking, the latter having the strongest association with foot function. Divergent validity, that is, the lack of or weak correlation between the test's score and variables, which in assumption have no impact on foot function, was verified by checking the correlation between FFI-RS outcome and DAS 28 (number of swollen and tender joints) from the questionnaire.

We chose DAS 28 for this purpose because it does not take into account the number of swollen and tender joints in the feet.

We also examined our research participants for the presence or absence of floor and ceiling effects. These effects show the proportion of patients who gain the lowest or highest possible scores and are considered to be present when more than 15% of the examined individuals achieve these scores.

Statistical calculations were conducted using SPSS v14. software. Statistical significance was established at the level of *p* < 0.05.

## 7. Results

The study involved 211 patients, 179 female and 32 male. The average age of patients was 58.9 ± 10.2 years. The characteristics of the research participants are shown in Table 1.

The average scores in the FFI-RS subscales were as follows: pain subscale 56.6 ± 16.8, stiffness subscale 59.1 ± 17.3, activity limitation subscale 54 ± 22.9, and social issues subscale 57.4 ± 22. The highest score (75.2 ± 17.7) was observed in difficulty subscale.

### 7.1. Reliability

Internal consistency was achieved at the high level of 0.95 using Cronbach's alpha test. The lowest score was achieved in activity limitation subscale (0.58). In the test-retest study, an interclass correlation coefficient (ICC) score of 0.81 was achieved for the whole questionnaire (Table 2).

### 7.2. Validity

Diagnostic validity was evaluated by checking the association between the FFI-RS scores and the ACR revised classification of functional status in RA. Participants in worse functional condition obtained higher FFI-RS questionnaire scores (*p* = 0.002). Analysis of the relation between FFI-RS total score and duration of RA showed the following: the longer the duration of RA, the higher the score obtained in FFI-RS, suggesting worse foot function. Moreover, the relation between pain location within the MTP joints and pain subscale of FFI-RS was analyzed. A statistically significant difference (*p* = 0.048) was observed between participants with and without MTP joint pain (Table 3).

Correlation of FFI-RS total score and outcomes in each of the subscales was studied consecutively with VAS pain scale, HAQ scale, and HAQ subscale concerning walking, as part of convergent validation. The low correlation was observed for the limitation subscale (Table 4).

In the divergent validity analysis, no correlation was observed between the numbers of swollen joints from the DAS 28 scale and FFI-RS scores. Weak or very weak correlation was indicated between the FFI-RS score and DAS 28, as well as between the FFI-RS score and the number of tender joints scores (Table 5). The presence of this correlation may be due to the fact that DAS 28 was designed for detecting systemic arthritis which is correlated in some extent with foot arthritis.

There were no floor or ceiling effects present for the summary of FFI-RS.

## 8. Discussion

To the best of our knowledge, this is the first adaptation and validation of FFI-RS into a language other than English. It needs to be stressed out that linguistic adaptation is a very important element of the validation process of every questionnaire. In the present study, the translation of FFI-RS from English into Polish was executed in such a way that the translated version would be as similar as possible to the original version in terms of psychometrics, construct, and language compatibility characteristics. As seen after back-translation, the obtained Polish version did not exhibit any significant differences from the original English version of the questionnaire.

The original version of the FFI is still being adapted into native languages in the international literature [17, 18]. The FFI-R was established over ten years priorly in response to criticism of the original version by many researchers and clinicians [9]. The FFI and FFI-R are widely used by both clinicians and researchers. Both indices have strong psychometrical properties, which is why they are commonly applied in the evaluation of the condition of the feet in patients suffering from RA and other diseases. Moreover, the FFI and FFI-R can be used to measure patients' recovery from surgical procedures or orthotic interventions in the foot and ankle [19–21]. However, as previously suggested, the weak point of the FFI is the fact that it was established without patients' contribution, which means that it may not express their opinion about foot lesions. Another drawback of the original FFI version is the fact that it was not based on any theoretical model. These weaknesses were eliminated with the establishment of the FFI-R version [1].

In our opinion, assessment using the FFI-R questionnaire, in combination with questions concerning life quality and psychosocial problems, may be more widely used by foot health experts in the fields of rheumatology, podiatry, and orthopedic medicine in Poland. It can be assumed that the revised version of the FFI-R will be more useful in measuring the patients' treatment outcomes.

While analyzing the literature dealing with the FFI-R, there were no reported significant differences in psychometrical parameters between FFI-RL (long form) and FFI-RS (short form). Both forms were shown to be equally qualified as measurement tools [1].

Considering economic issues and practicality in the patient care management process, we decided to adapt and validate the FFI-RS questionnaire into Polish.

Our results confirmed the accuracy of the FFI-RS questionnaire in terms of achieving a high level of 0.95 in Cronbach's alpha test. The highest score was achieved in the difficulty subscale (0.93) and the lowest score in the activity limitation subscale (0.58) (Table 2).

A lower score in the activity limitation subscale compared to other subscales may be due to the fact that this subscale has only three questions. Moreover, two of the questions were answered as “not applicable” (numerical 5 was selected), which means that participants had not performed those particular activities. This observation was taken into consideration while summing up the final result of the subscale.

In the diagnostic validity examination, statistically higher results were obtained in patients with pain located within the MTP joints, long-lasting duration of the disease, and worse functional condition, indicating impaired foot function.

The outcomes obtained during convergent and divergent validity checks were in line with the authors' assumptions.

The DAS 28 scale, which assesses the wrist, hand, elbow, shoulder, and knee, but not foot and ankle [22, 23] joint's function, is currently used to evaluate the disease activity of patients suffering from RA. However, it needs to be highlighted that the American College of Rheumatology and the European League Against Rheumatism ACR/EULAR diagnostic criteria, published in 2010, take into consideration the saccadic, ankle joint and MTPs joints, excluding the first MTP joint [10, 24], which confirms the importance and validity of the FFI-R application as an important and comprehensive diagnostic tool.

A significant finding from previous studies was that 15% of patients suffering from RA developed the first symptoms of the disease in their feet [4]. As the disease progressed, more patients suffered damage in the joints of the feet. The authors of that study concluded that there is a need among clinicians for wider use of questionnaires for evaluating the feet. These measures are practical, inexpensive, and not time-consuming. These measures could evaluate patients' foot function and monitor the effects of physical therapy.

The limitation of the present study is the fact that the responsiveness to the changes in the FFI-RS questionnaire was not checked. An ongoing study of our group on the usefulness of FFI-RS (short form) will evaluate the physiotherapeutic procedures among patients with rheumatological disorders located within feet. Another limitation was the fact that the FFI-RS PL was studied in patients among older RA patients, drawn from a single hospital (monocentric). It is possible that the outcomes of the DAS 28 and FFI-RS subscales may be different in a younger population, who have more acute/active joint swelling. Also patients without RA may present different results in FFI-RS scores. Next limitation is the fact that we do not know accurate target population of the hospital (rural and city environments). The study should be continued with consideration of cultural, socioeconomic, and language differences in Polish population.

## 9. Conclusion

The Polish version of FFI-RS-PL is an important evaluating tool and can be applied in clinical and epidemiological studies among Polish-speaking patients suffering from RA. FFI-RS-PL research process should be conducted in other patient groups.

## Figures and Tables

**Table 1:** Characteristics of the research participants.

Age (years)	58.9 ± 10.21
Sex	Female 179/male 32
The disease duration (years)	15.9 ± 15.84
BMI	27.53 ± 4.99
DAS 28	4.33 ± 0.97
VAS (mm)	58.04 ± 16.79
HAQ	2.44 ± 1.36

All data are expressed as mean ± SD; BMI: Body Mass Index; DAS28: Disease Activity Score 28; VAS: Visual Analog Scale; HAQ: Health Assessment Questionnaire.

**Table 2:** Reliability: Cronbach's alpha value and intraclass correlation coefficient.

FFI-RS	Cronbach's alpha	ICC
FFI-RS total	0.95	0.81
FFI-RS pain	0.81	0.84
FFI-RS stiffness	0.85	0.81
FFI-RS difficulty	0.93	0.79
FFI-RS activity limitation	0.58	0.79
FFI-RS social issues	0.85	0.78

FFI-RS: Foot Function Index-Short Form; ICC: intraclass correlation coefficient.

**Table 3:** Diagnostic validity.

ACR revised classification of functional status in RA	1 (*n* = 13)	2 (*n* = 127)	3 (*n* = 68)	4 (*n* = 3)	*p* = 0,002 Kruskal-Wallis Test
FFI-RS (mean)	51.1 ± 15	62.2 ± 15.5	65.6 ± 13.5	81.6 ± 11.1	

Disease duration in years	1–5 (*n* = 33)	6–10 (*n* = 45)	11–20 (*n* = 75)	>20 (*n* = 58)	*p* < 0,0001 Kruskal-Wallis Test
FFI-RS	55.4 ± 16.1	58.1 ± 17.7	64.5 ± 14.3	68.7 ± 10.9	

MTP joint pain	No (*n* = 135)		Yes (*n* = 76)		*p* = 0,048 Mann-Whitney Test
FFI-RS, pain	54.6 ± 16.7		60.2 ± 16.5		

Results are expressed as mean (SD); FFI-RS: Foot Function Index-Short Form.

**Table 4:** Convergent validity.

FFI-RS	VAS	HAQ	HAQ walking
FFI-RS total	0.54^*∗∗*^	0.61^*∗∗*^	0.51^*∗∗*^
FFI-RS pain	0.50^*∗∗*^	0.41^*∗∗*^	0.32^*∗∗*^
FFI-RS stiffness	0.52^*∗∗*^	0.50^*∗∗*^	0.43^*∗∗*^
FFI-RS difficulty	0.47^*∗∗*^	0.61^*∗∗*^	0.52^*∗∗*^
FFI-RS activity limitation	0.32^*∗∗*^	0.37^*∗∗*^	0.31^*∗∗*^
FFI-RS social issues	0.34^*∗∗*^	0.52^*∗∗*^	0.43^*∗∗*^

^*∗∗*^Significant correlation (*p* < 0.01; 2-tailed).

**Table 5:** Divergent validity.

FFI-RS	Number of swollen joints	Number of tender joints	DAS 28
FFI-RS total	0.08	0.26^*∗∗*^	0.26^*∗∗*^
FFI-RS pain	0.08	0.19^*∗∗*^	0.23^*∗∗*^
FFI-RS stiffness	0.11	0.24^*∗∗*^	0.26^*∗∗*^
FFI-RS difficulty	0.08	0.25^*∗∗*^	0.26^*∗∗*^
FFI-RS activity limitation	0.01	0.19^*∗∗*^	0.10
FFI-RS social issues	−0.01	0.17^*∗*^	0.16^*∗*^

^*∗∗*^Significant correlation (*p* < 0.01; 2-tailed). ^*∗*^Significant correlation (*p* < 0.05; 2-tailed).

## Supplementary Materials

Supplementary Materials The Supplementary Materials include 2 files: (1) Foot Function Index-Revised Short Form-Polish Version (FFI-RS-PL). (2) Revised Foot Function Index (FFI-R) Short Form-English Version.
