# Supplementary material for: Adaptation and Validation of the Foot Function Index-Revised Short Form into Polish
Source: Biomed Res Int. 2017 Nov 27;2017:6051698. doi: 10.1155/2017/6051698 (PMC5733175; doi:10.1155/2017/6051698)
Supplement: Supplementary Materials — The Supplementary Materials include 2 files: (1) Foot Function Index-Revised Short Form-Polish Version (FFI-RS-PL). (2) Revised Foot Function Index (FFI-R) Short Form-English Version. [file 6051698.f1.docx]

| **Kwestionariusz oceny funkcjonalności stóp- wersja skrócona (FFI-RS-PL)**  Pacjent…………………………………………………………. data………...  **BÓL**  ***PROSZĘ PRZECZYTAĆ PRZED UDZIELANIEM ODPOWIEDZI:***   - Poniższe pytania odnoszą się do nasilenia BÓLU stóp. Proszę zaznaczyć w każdej odpowiedzi nasilenie bólu jaki odczuwał/a Pan/Pani w ciągu ostatniego tygodnia podczas różnych sytuacji. Brak bólu proszę zaznaczyć jako 1, natomiast jeżeli ból był silny proszę zakreślić numer 4. - Jeżeli pewne pytania nie dotyczą Pana/Pani proszę zakreślić numer 5. - Proszę uzupełnić odpowiedzi na wszystkie pytania.  1. **JAK SILNY BYŁ BÓL STÓP PODCZAS OSTATNIEGO TYGODNIA:**  \|  \| brak bólu \| słaby ból \| średni ból \| silny ból \|  \| \| --- \| --- \| --- \| --- \| --- \| --- \| \| 1. Przed rannym wstaniem? 2. Przy pierwszym staniu bez butów? 3. Kiedy stałaś/eś po włożeniubutów? 4. Podczas chodzenia w butach? 5. Podczas stania mając buty ze specjalnymi wkładkami? 6. Podczas chodzenia w butach ze specjalnymi wkładkami? 7. Pod koniec zwykłego dnia? \| 1  1  1  1  1  1  1 \| 2  2  2  2  2  2  2 \| 3  3  3  3  3  3  3 \| 4  4  4  4  4  4  4 \| 5-brak wkładek  5-brak wkładek \|   **SZTYWNOŚĆ STÓP**  ***PROSZĘ PRZECZYTAĆ PRZED UDZIELANIEM ODPOWIEDZI:***   - Poniższe pytania odnoszą się do nasilenia SZTYWNOŚCI (*NIE BÓLU*) stóp. Proszę zaznaczyć w każdej odpowiedzi nasilenie sztywności jakie odczuwał/a Pan/Pani w ciągu ostatniego tygodnia podczas różnych sytuacji. Brak sztywności proszę zaznaczyć jako 1, natomiast w przypadku odczuwania dużej sztywności proszę zakreślić numer 4. - Jeżeli pewne pytania nie dotyczą Pana/Pani proszę zakreślić numer 5. - Proszę uzupełnić odpowiedzi na wszystkie pytania.  1. **JAK DUŻA BYŁA SZTYWNOŚĆ STÓPPODCZAS OSTATNIEGO TYGODNIA:**  \|  \| brak sztywności \| słabo nasilona sztywność \| średnio nasilona sztywność \| duża sztywność \|  \| \| --- \| --- \| --- \| --- \| --- \| --- \| \| 1. Przed rannym wstaniem? 2. Przy pierwszym staniu bezbutów? 3. Przy pierwszym chodzeniu bez butów? 4. Kiedy stałaś/eś po włożeniubutów? 5. Podczas chodzenia w butach? 6. Podczas chodzenia w butach ze specjalnymi wkładkami? 7. Przed pójściem spać w nocy? \| 1  1  1  1  1  1  1 \| 2  2  2  2  2  2  2 \| 3  3  3  3  3  3  3 \| 4  4  4  4  4  4  4 \| 5-brak wkładek \|   **UTRUDNIENIA**  ***PROSZĘ PRZECZYTAĆ PRZED UDZIELANIEM ODPOWIEDZI:***   - Poniższe pytania odnoszą się do stopnia trudności wykonywania czynności związanego z problemami z powodu stóp. Proszę zaznaczyć w każdej odpowiedzi stopień trudności jaki występował u Pana/Pani w ciągu ostatniego tygodnia podczas wykonywania różnych czynności. Brak utrudnień proszę zaznaczyć jako 1, natomiast duże utrudnienia proszę zakreślić numer 4. - Proszę uzupełnić odpowiedzi na wszystkie pytania  1. **JAK POWAŻNE BYŁY UTRUDNIENIA SPOWODOWANE PRZEZ PROBLEMY STÓP PODCZAS OSTATNIEGO TYGODNIA:**  \|  \| brak utrudnień \| łagodne utrudnienia \| średnie utrudnienia \| duże utrudnienia \| \| --- \| --- \| --- \| --- \| --- \| \| 1. Chodząc poza domem po nierównym podłożu? 2. Po przejściu około 1000 m? 3. Wchodząc po schodach? 4. Schodząc po schodach? 5. Stojąc na palcach? 6. Podczas noszenia bądź podnoszenia ciężaru powyżej 2 kilogramów? 7. Wstając z krzesła? 8. Idąc szybkim tempem? 9. Biegnąc? 10. W utrzymywaniu równowagi? 11. Chodząc z przyrządami wspomagającymi (laska, kula,balkonik)? \| 1  1  1  1  1  1  1  1  1  1  1 \| 2  2  2  2  2  2  2  2  2  2  2 \| 3  3  3  3  3  3  3  3  3  3  3 \| 4  4  4  4  4  4  4  4  4  4  4 \|   **OGRANICZENIA AKTYWNOŚCI**  ***PROSZĘ PRZECZYTAĆ PRZED UDZIELANIEM ODPOWIEDZI:***   - Proszę zakreślić liczbę wskazującą jak często występowało u Pana/Pani ograniczenie aktywnościzwiązane z problemem stóp podczas ostatniego tygodnia. - Np., przy pytaniu jak często problem stóp ograniczał Twoją aktywność poza domem?, proszę zakreślić 1- jeżeli nigdy, natomiast 4 - jeżeli zawsze. - Jeżeli pewne pytania nie dotyczą Pana/Pani proszę zakreślić numer 5. - Proszę uzupełnić odpowiedzi na wszystkie pytania.  1. **JAK CZĘSTO WYSTĄPIŁA DANA SYTUACJA PODCZAS OSTATNIEGO TYGODNIA:**  \|  \| nigdy \| rzadko \| często \| zawsze \|  \| \| --- \| --- \| --- \| --- \| --- \| --- \| \| 1. Zostawałaś/eś w domu przez większączęść dnia z powodu problemów ze stopami? 2. Problem stóp ograniczał Twoją aktywność poza domem? 3. Problem stóp ograniczał Twoją aktywność sportową? \| 1  1  1 \| 2  2  2 \| 3  3  3 \| 4  4  4 \| 5-nie byłeś/aś  5-brak sportu \|   **SPRAWY SOCJALNE**  ***PROSZĘ PRZECZYTAĆ PRZED UDZIELANIEM ODPOWIEDZI:***   - Proszę zakreślić liczbę wskazującą jak często towarzyszyło Panu/Pani dane uczucie z powodu problemów ze stopami, podczas ostatniego tygodnia. - Np., przy pytaniu jak często problem stóp powodowałograniczenia życia towarzyskiego ?, proszę zakreślić 1- jeżeli nigdy, 4- jeżeli zawsze. - Jeżeli pewne pytania nie dotyczą Pana/Pani proszę zakreślić numer 5. - Proszę uzupełnić odpowiedzi na wszystkie pytania.  1. **JAK CZĘSTO ODCZUWAŁ/A PAN/I PODCZAS OSTATNIEGO TYGODNIA:**  \|  \| nigdy \| rzadko \| często \| zawsze \|  \| \| --- \| --- \| --- \| --- \| --- \| --- \| \| 1. Zawstydzenie z powodu noszonych butów? 2. Złe samopoczucie z powodu problemów ze stopami? 3. Ograniczenia życia towarzyskiego z powodu problemów ze stopami? 4. Trudności w uczestnictwie w życiu towarzyskim z powodu noszonego obuwia? 5. Obciążenie koniecznością przyjmowania leków w celu radzenia sobie z bólem? 6. Obawę o zaniedbanie pracy w domu? \| 1  1  1  1  1  1 \| 2  2  2  2  2  2 \| 3  3  3  3  3  3 \| 4  4  4  4  4  4 \| 5  5  5 \|   **KOMENTARZ KOŃCOWY**  Proszę o komentarz na temat:   1. Czy polecenia były jasno sformułowane? 2. Czy było pytanie, na które miał/a Pan/Pani trudności ze zrozumieniem? 3. Czy było niejasne pytanie, jeżeli tak to które? 4. Czy z powodu któregoś pytania czuł/a się Pan/Pani niekomfortowo? Jeżeli tak, które to pytanie i dlaczego? 5. Czy jest jakiś aspekt, który nie został poruszony w ankiecie, a który warto do niej dodać? Jeżeli tak, jaki to aspekt? 6. Czy miał/a Pan/Pani jakieś problemy związane z kwestionariuszem, o których chciałbyś/chciałabyś wspomnieć? Jeżeli tak, to jakie to problemy?   **Dziękujemy za udział w tym badaniu.**  **Wynik**  Ból .........  Sztywność .........  Utrudnienia ..........  Aktywność .........  Sprawy socjalne .........  Całkowity .........  **Revised FOOT FUNCTION INDEX (FFI-R) Short Form**  Subject ID: [_____]_____]_____]______] [Date: [___]___] / [___]___] / [___]___]___] PAIN **Pleasereadbeforeanswering.**   - Please circle the number that indicates how bad your foot pain was in each of the following situations during the past week. - For example, when asked how severe your foot pain was at its worst, if you feel “No pain,” circle the number 1 and if you felt “Severe pain,” circle the number 4. - If, for some items, the question does not apply, circle the number 5. - Please provide an answer for every item.   1. DURING THE PAST WEEK, HOW SEVERE WAS YOUR FOOT PAIN:  No Mild Moderate Severe  Pain pain pain pain  1. Before you get up in the morning? . . . . . . . . . . . . 1 2 3 4  2. When you first stood without shoes? . . . . . . . . . 1 2 3 4  3. When you stood wearing shoes? . . . . . . . . . . . . . . 1 2 3 4  4 When you walked wearing shoes? . . . . . . . . . .. . . 1 2 3 4  5. When you stood wearing custom shoe inserts? . . 1 2 3 4 5 = do not use  inserts  6. When you walked wearing custom shoe inserts? . 1 2 3 4 5= do not use  inserts  7. At the end of a typical day? . . . . . . . . . . . . . . . . . 1 2 3 4    Subject ID: [_____]_____]_____]______]  **STIFFNESS**  **Pleasereadbeforeanswering.**   - Please circle the number that indicates how bad your foot stiffness was in each of the following situations during the past week. - For example, when asked how severe your foot stiffness was at its worst, if you feel “No stiffness,” circle the number 1 and if you felt “Severe stiffness,” circle the number 4. - If, for some items, the question does not apply, circle the number 5. - Please provide an answer for every item.   1. DURING THE PAST WEEK, HOW SEVERE WAS YOUR FOOT STIFFNESS:  No Mild Moderate Severe  stiffness stiffness stiffness stiffness  8. Before you get up in the morning? . . . . . . . . . . . . 1 2 3 4  9. When you stood without shoes? . . . . . . . . . . . . . 1 2 3 4  10. When you walked without shoes? . . . . . . . . . . . 1 2 3 4  11. When you stood wearing shoes? . . . . . . . . . . . . . . 1 2 3 4  12. When you walked wearing shoes? . . . . . . . . . .. . . 1 2 3 4  13. When you walked wearing custom shoe inserts? . 1 2 3 4  14. Before you went to sleep at night? . . . . . . . . . . . 1 2 3 4    Subject ID: [_____]_____]_____]______] DIFFICULTY **Pleasereadbeforeanswering.**   - Please circle the number that indicates how much difficulty you had performing each activity because of your foot problems during the   past week.   - For example, when asked how much difficulty your foot problems caused when walking around the house, if you had “No difficulty,” circle the number 1 and if it was ” Severe difficulty,” circle the number 4. - If, for some items, the question does not apply, circle the number 5. - Please provide an answer for every item.   2. DURING THE PAST WEEK, HOW MUCH DIFFICULTY DID YOUR FOOT PROBLEMS CAUSE YOU:  No Mild Moderate Severe difficulty difficulty difficulty difficulty    15. Walking outside on uneven ground? . . . . . . 1 2 3 4  16. Walking four or more blocks? . . . . . . . . . . . 1 2 3 4  17. Climbing stairs? . . . . . . . . . . . . . . . . . . . . . . 1 2 3 4  18. Descending stairs? . . . . . . . . . . . . . . . . . . . . 1 2 3 4  19. Standing on tip toes? . . . . . . . . . . . . . . . . . . 1 2 3 4  20. When you carried or lifted objects  weighing more than five pounds? . . . . . . . . 1 2 3 4  21. Getting out of a chair? . . . . . . . . . . . . . . . . . 1 2 3 4  22. Walking fast? . . . . . . . . . . . . . . . . . . . . . . . 1 2 3 4  Subject ID: [_____]_____]_____]______]  3. (cont.) DURING THE PAST WEEK, HOW MUCH DIFFICULTY DID YOUR FOOT PROBLEMS CAUSE YOU:  No Mild Moderate Severe difficulty difficulty difficulty difficulty    23. Running? . . . . . . . . . . . . . . . . . . . . . . . . . . . 1 2 3 4  24. Keeping your balance? . . . . . . . . . . . . . . . . 1 2 3 4  25. Walking with assistive devices? . . . . . . . . 1 2 3 4    Subject ID: [_____]_____]_____]______] ACTIVITY LIMITATION **Pleasereadbeforeanswering.**   - Please circle the number that indicates how often you performed each of these activities in the past week because of your feet. - For example, when asked how often you used a cane indoors because of foot problems, if you used one “None of the time,” circle the number 1 and if you used one “All of the time,” circle the number 4. - If, for some items, the question does not apply, circle the number 5. - Please provide an answer for every item.   4. DURING THE PAST WEEK, HOW MUCH OF THE TIME DID YOU:  None Some Most All  of the time of the time of the time of the time  26. Stay indoors most of the day because of  foot problems? . . . . . . . . . . . . . . . . . . . . . . . . . . 1 2 3 4  27. Limit your outdoor activities because of  foot problems? . . . . . . . . . . . . . . . . . . . . . . . . . 1 2 3 4 5= No outdoor  activities  28. Limit your leisure/sport activities  because of foot problems? . . . . . . . . . . . . . . . 1 2 3 4 5 = Do not play  sports    Subject ID: [_____]_____]_____]______] SOCIAL ISSUES **Pleasereadbeforeanswering.**   - Please circle the number that indicates how often you experienced the following feelings in the past week because of your feet. - For example, when asked how often you felt a fear of falling because of foot problems, if you felt fear “None of the time,” circle the number 1 and if you felt fear “All of the time,” circle the number 4. - If, for some items, the question does not apply, circle the number 5. - Please provide an answer for every item.   5. DURING THE PAST WEEK, HOW MUCH OF THE TIME DID YOU EXPERIENCE:  None of Some Most All of  the time the time the time the time    29. Embarrassment due to footwear? . . . . . . . . . . . 1 2 3 4  30. Feeling awful because of foot problem? . . . . . . 1 2 3 4  31. Limit social activities due to foot problems? . . 1 2 3 4  32. Difficulty participating in social activities  due to footwear? . . . . . . . . . . . . . . . . . . . . . . . 1 2 3 4 ______  33. Burden of taking medication to control  foot pain? . . . . . . . . . . . . . . . . . . . . . . . . . . . . 1 2 3 4 ______  34. Concern about limited work around the house?. 1 2 3 4 ______  **SUBJECT COMMENTS:**  Please comment about:   1. Were the directions clear? 2. Were any of the questions difficult to understand? 3. Were any of the questions unclear? If yes, which ones and why? 4. Did any of the questions make you uncomfortable? If yes, which ones and why? 5. Are there any issues about your feet that were not asked or that you would add to the questionnaire? If yes, which issues? 6. Did you have any problems with this questionnaire that you would like to mention? If yes, which problems?   Thank you for participating in this study.  Pain score: ______  Stiffness score: ______  Difficulty score: ______  Activity score: ______  Social score: ______  Cumulative score: ______ |
| --- | --- | --- | --- | --- | --- | --- | --- | --- | --- | --- | --- | --- | --- | --- | --- | --- | --- | --- | --- | --- | --- | --- | --- | --- | --- | --- | --- | --- | --- | --- | --- | --- | --- | --- | --- | --- | --- | --- | --- | --- | --- | --- | --- | --- | --- | --- | --- | --- | --- | --- | --- | --- | --- | --- | --- | --- | --- | --- |
